# Genetic association study of common variants in *TGFB1* and *IL-6* with developmental dysplasia of the hip in Han Chinese population

**DOI:** 10.1038/s41598-017-11185-1

**Published:** 2017-08-31

**Authors:** Wenlong Ma, Zhuqing Zha, Ke Chen, Honggan Chen, Yixin Wu, Jianbing Ma, Sixiang Zeng, Liqiang Zhi, Shuxin Yao

**Affiliations:** 1 Department of Hip Injury and Disease, Orthopedic Hospital of Henan Province, Qiming South Road No.82, Luoyang, Henan China; 2 Department of Joint Surgery, Honghui Hospital, Xi’an Jiaotong University Health Science Center, Xi’an, Shaanxi China; 3 The Third Clinical Medical College of Guangzhou, University of Traditional Chinese Medicine, Guangzhou, Guangdong, China; 4 Department of Orthopedics, the First Affiliated Hospital, Xi’an Jiaotong University, Xi’an, Shaanxi China

## Abstract

Developmental dysplasia of the hip (DDH) is a congenital or developmental deformation or misalignment of the hip joint that is affected by environmental and genetic factors. Recently, polymorphisms in both *TGFB1* and *IL-6* have been identified as being significantly associated with hip osteoarthritis in Caucasians. In this study, we conducted a case-control study involving 4,206 Han Chinese individuals to investigate the effects of *TGFB1* and *IL-6* on the disease status and severity of DDH. A total of 32 single-nucleotide polymorphisms (SNPs) were selected to ensure coverage of the two genetic loci. We found SNP rs1800470 in *TGFB1* (OR = 1.255, *P* = 0.0004) and rs1800796 (OR = 0.84, *P* = 0.0228) in *IL-6* to be significantly associated with DDH in this cohort. Further haplotype-based analysis replicated this significant result. Another SNP in *IL-6*, rs1800796, showed a marginally significant association with DDH. As a non-synonymous SNP, rs1800470 alters the amino acid sequence of the polypeptide encoded by *TGFB1*; however, bioinformatics analyses revealed that this SNP has limited functional significance. No significant results were obtained in an association study focusing on the severity of DDH and epistasis analysis. Our findings support an important role for *TGFB1* in the risk of DDH. Further research is needed to validate the weak association between rs1800796 in *IL-6* and DDH.

## Introduction

Developmental dysplasia of the hip (DDH) is a congenital or developmental deformation or misalignment of the hip joint that endangers skeletal development in children^1^. As one of the most common skeletal congenital anomalies, DDH affects 1 in 1000 live births in the Caucasian population^2^. In China, the incidence rate of DDH ranges from 1 to 10 in 1000 live births^3, 4^. Overall, this condition is five times more frequent in females than in males^5^. The severity of DDH ranges from a mild form of acetabular dysplasia to a moderate form of subluxation of the hips and even to the severe form of complete dislocation of the hips^1^. As a complex developmental disorder, DDH is affected by both environmental and genetic factors. Early gene-association mapping studies have identified several susceptibility loci for DDH. For example, a genome-wide linkage scan based on a Japanese family with acetabular dysplasia (a mild form of DDH) and osteoarthritis identified a peak region at chromosome 13q22^6^. Although no genome-wide association study focusing on DDH has been reported to date, but several well-designed candidate gene-based association mapping studies have identified several genes, including *CX3CR1* ^7^, *DKK1* ^8^, *HOXD9* ^9^ and *PAPPA2* ^10^. In addition to studies focusing on disease status, some early analyses have focused on the severity of DDH, identifying polymorphism markers in such genes as *ASPN* ^11^, *GDF5* ^12^, *HOXB9* ^13^, and *TBX4* ^14^ as being significantly associated with DDH severity.

*TGFB1* encodes transforming growth factor beta 1 (TGF-β1), a member of the transforming growth factor beta superfamily of cytokines^15^. As a secreted protein, TGF-β1 performs many cellular functions, including the control of cell growth, proliferation, differentiation and apoptosis^15^. The gene *IL-6* encodes an interleukin that can act as both a pro-inflammatory cytokine and an anti-inflammatory myokine^16^. Both TGF-β1 and IL-6 are pro-inflammatory cytokines involved in the pathogenesis of hip osteoarthritis^17^, which is a major consequence of DDH in adulthood, and both proteins are also involved in the bone remodelling process^18^. Dickinson *et al*. investigated the mRNA levels of *TGFB1* in different tissues, and the findings suggested a role in growth and differentiation in cartilage, endochondral and membrane bone, and skin^19^. Furthermore, polymorphisms in *TGFB1* and *IL-6* have been identified as significantly associated with hip osteoarthritis in studies based on Caucasian populations^20, 21^. However, most of these early studies suffered from the lacks of replication and insufficient statistical power which may be due to the sample size and small effect size or a combination of both.

In this study, we conducted a case-control study based on 4,206 Han Chinese subjects to investigate the potential effects of *TGFB1* and *IL-6* on the disease status and severity of DDH in this population. Thirty-two single-nucleotide polymorphisms (SNPs) were selected to ensure coverage of the two genetic loci. The main purpose of our study was to replicate some existing results in Han Chinese population. Our study explored specific linkage disequilibrium (LD) structure of Han Chinese population on *TGFB1* and *IL-6*, and identified Han Chinese specific connection between the polymorphisms within *TGFB1* and *IL-6* and DDH.

## Methods

### Subjects

A total of 373 patients (294 females and 79 males) with DDH and 1,115 controls (883 females and 232 males) recruited from Luoyang Orthopaedic Hospital were included as discovery samples, and 691 patients (583 females and 108 males) with DDH and 2,027 controls (1,701 females and 326 males) enrolled from Xi’an Honghui Hospital and the First Affiliated Hospital of Xi’an Jiaotong University were included as validation samples. The study subjects recruited in this study were unrelated Han Chinese individuals from the city of Luoyang in Henan Province and Xi’an in Shaanxi Province. Patients were diagnosed by medical examination with radiographic evidence, and all had unilateral or bilateral DDH. The severity of DDH was defined according to three grades: instability; subluxation; dislocation. The controls were identified by a detailed inquiry of their medical history and physical examination, and none had a history or symptoms of DDH. Subjects with any systemic syndrome were excluded from our study. Written informed consent was obtained from the study subjects. Related clinical characteristics and demographic data for the subjects are summarized in Table 1. The study protocol conformed to the ethical guidelines of the 1975 Declaration of Helsinki and was approved by the Ethics Committee of Luoyang Orthopaedic Hospital, Xi’an Honghui Hospital and the First Affiliated Hospital of Xi’an Jiaotong University.

**Table 1:** Characteristics of DDH patients and healthy controls.

Characteristics	Discovery (N = 1,488)		Validation (N = 2,718)	
	DDH patients	Healthy controls	DDH patients	Healthy controls
Number	373	1,115	691	2,027
Age, mean ± SD (months)	23.6 ± 21.4	70.3 ± 44.4	20.8 ± 15.6	66.7 ± 31.6
Male/Female	79/294	232/883	108/583	326/1,701
Acetabular dysplasia	18		42	
Unilateral	12	NA	28	NA
Bilateral	6	NA	14	NA
Subluxation of the hips	82		131	
Unilateral	62	NA	102	NA
Bilateral	20	NA	29	NA
Dislocation of the hips	293		518	
Unilateral	186	NA	378	NA
Bilateral	87	NA	140	NA

DDH: developmental dysplasia of the hips; SD: standard deviation; NA: not available.

### SNP Selection and Genotypingj

**Figure 1:**
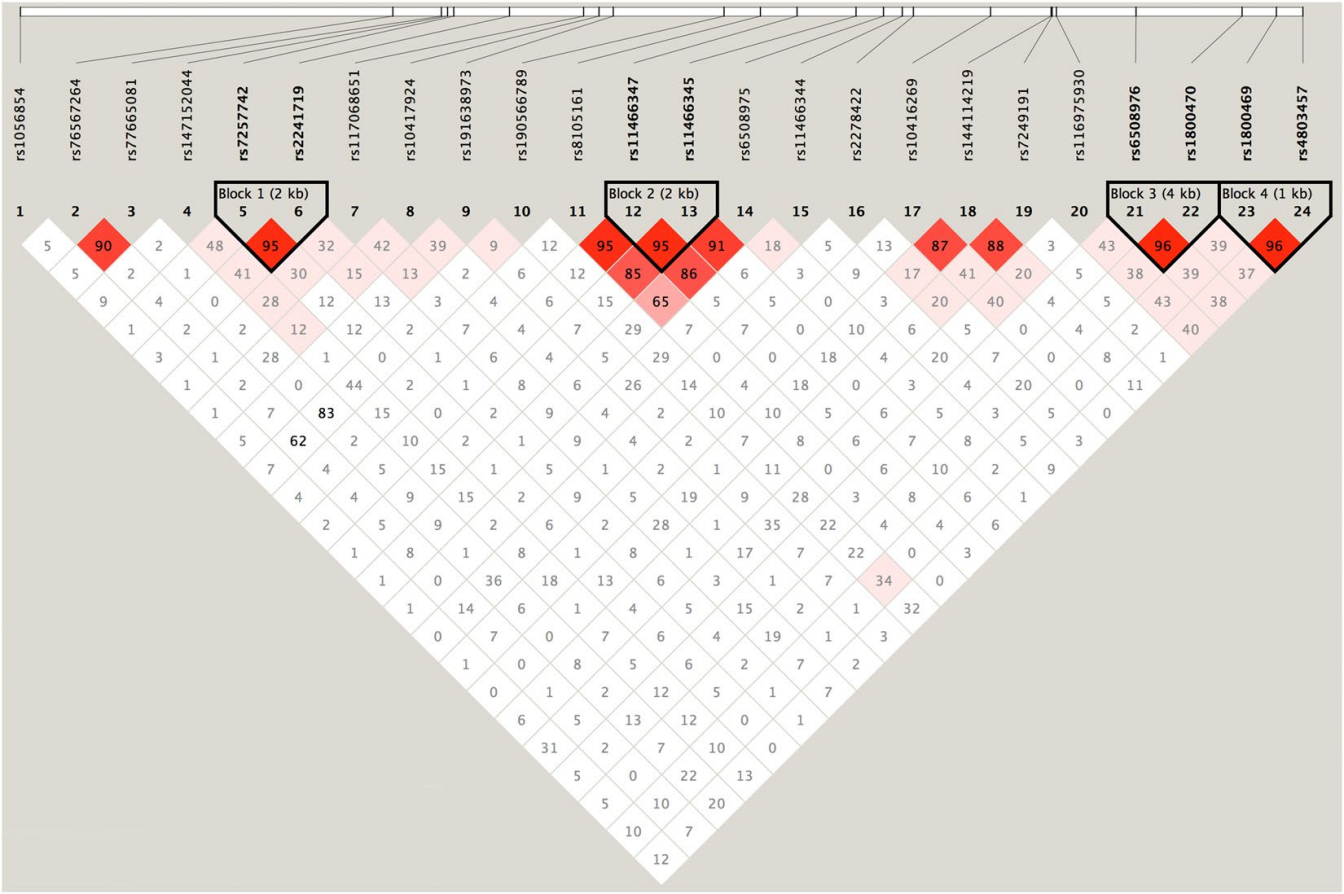
Linkage disequilibrium (LD) patterns of the region around the *TGFB1* gene in the Han Chinese population. The LD blocks are indicated as shaded matrices, and four LD blocks were found.

Using 1000 Genomes CHB (Han Chinese in Beijing) data^22^, we selected tagging SNPs for all SNPs in the *IL6* and *TGFB1* gene regions with minor allele frequencies (MAFs) ≥ 0.01. Haploview was utilized for the SNP selection process; SNP tagging was performed by Tagger, a built-in algorithm of Haploview^23^. The threshold we used for tagging was *r* ^*2*^ *≤* 0.8, which is also the default setting of Tagger. Finally, 32 tag SNPs covering the *IL6* (8 tag SNPs) and *TGFB1* (24 tag SNPs) gene regions were included in the study (Supplemental Table S1). Haploview was also utilized to visualize LD structure^24, 25^. The LD structure based on the data for the discovery stage is shown in Fig. 1 and Supplemental Figure S1.

Genomic DNA was isolated from peripheral blood using a Tiangen DNA extraction kit (Tiangen Biotech Co. Ltd, Beijing, China) according to the manufacturer’s protocol. SNP genotyping was performed using a Sequenom MassARRAY platform with iPLEX GOLD chemistry (Sequenom, San Diego, CA, USA) based on the manufacturer’s protocols. The results were processed using Sequenom Typer 4.0 software^26^, and genotype data were generated from the samples. Genotyping was conducted by laboratory personnel blinded to the case-control status, and the genotyping results, data entry and statistical analyses were independently reviewed by two authors. We randomly re-performed the analysis on 5% of the sample, with a concordance of 100%.

### Statistical Analyses

MAFs and Hardy-Weinberg equilibrium of the genotyped SNPs were calculated and tested in the discovery stage using Plink^27^. Logistic models were fit to assess potential associations between the 32 selected SNPs and DDH disease status in the discovery stage, including age and sex as covariates for each model. Genotypes of the SNPs were coded in three models: additive, dominant and recessive. For the additive model, the genotype was coded as 0, 1, or 2 for the three genotypes AA, Aa, and aa (where a is the minor allele), respectively. For the dominant model, the genotype was coded as 0, 1, or 1; for the recessive model, the genotype was coded as 0, 0, or 1. For SNPs that showed nominal significance in the discovery stage, genotyping of subjects was conducted in the validation stage. In addition, we also included those SNPs that were in strong LD (*r* ^2^ > 0.9) with nominally significant SNPs in the validation stage. To enhance the statistical power, we tested the selected markers using the combined sample from both the discovery and validation stages. Logistic models were also fit to evaluate the potential effects of selected markers, as in the discovery stage; in addition to age and sex, we included the batch number of sample recruitment as a covariate to adjust for this potential confounding factor. Logistic models were fit and tested using Plink^27^. In addition to the single-marker-based analysis, we conducted haplotype-based analysis using data from the discovery stage. LD blocks based on the 32 SNPs with the sample from the discovery stage were constructed using methods proposed by Gabriel *et al*.^28^. Essentially, 95% confidence bounds on D’ are generated and each comparison is considered “strong LD” when the confidence bounds have an upper bound ≥ 0.98 and a lower bound ≥ 0.7; a block is created if 95% of informative comparisons are “strong LD”. Haplotype based analysis can be more powerful than single marker based association analysis especially when the haplotypes were formed by several highly correlated SNPs. Therefore we tested the haplotypes of the LD blocks their significance using Plink^27^.

To evaluate the potential effects of different types of DDH on our significant findings, we separately conducted single-marker-based analyses using DDH samples of different types, i.e., acetabular dysplasia, subluxation of hips and dislocation of hip. In addition, to test whether significant SNPs are also indicators of the severity of the disorder, we dichotomized our DDH cases as two categories: the severe form, which included samples with dislocation of the hips only, and the mild to moderate form, which included samples of acetabular dysplasia and subluxation of the hips. We then performed logistic regression for SNPs that were significantly associated with the disease status of DDH. To assess potential gene-by-gene interaction between *IL6* and *TGFB1*, we also conducted interaction analyses using logistic models by adding a multiple term (sex, age and stage number were also included in the models as covariates). Both the stratification and case-only severity analyses were performed using Plink^27^, and interaction analyses were performed using R project^29^. Bonferroni corrections were applied to all analyses described above to adjust for multiple comparisons.

### Bioinformatics Analyses

Several bioinformatic databases and tools were utilized to evaluate the functional significance of the targeted SNPs. The open bioinformatics platform Galaxy was utilized to extract basic information for selected SNPs^30^. RegulomeDB was employed to evaluate the potential functional significance of selected SNPs^31^. Analyses using Polyphen2^32^ and SIFT^33^ were performed to examine potential effects of non-synonymous SNPs on protein function. In addition, we used the protein-protein interaction database STRING^34^ to explore features of the gene-gene networks surrounding our candidate genes.

## Results

### Association Analyses

Single-marker-based association analysis identified 2 of the 32 SNPs as nominally significant in samples during the discovery stage (rs1800470, OR = 1.37, *P* = 0.0023, *TGFB1*; rs1800796, OR = 0.79, *P* = 0.0427, *IL6*). Four SNPs, including two other in strong LD with the significant SNPs, were genotyped in the validation sample. Analyses based on the combined sample from both the discovery and validation stages showed that two SNPs, rs1800470 (OR = 1.255, P = 0.0004) and rs1800796 (OR = 0.84, P = 0.0228), were significantly associated with the disease status of DDH. The results of the 4 SNPs genotyped based on the additive model in the combined sample are shown in Table 2. The complete results of the single-marker-based analyses in the discovery stage are summarized in Supplemental Table S2. Four LD blocks were constructed based on the 32 selected markers in the discovery stage (Fig. 1 and Supplemental Figure S1). Haplotype analysis identified a 2-SNP haplotype as significantly associated with the disease status of DDH (rs6508976-rs1800470, *P* = 1.65 × 10^−26^), and this finding confirmed the results of the single-marker-based analyses (Supplemental Table S3).

**Table 2:** Single-marker-based analyses of 4 SNPs that were genotyped in samples (additive model).

CHR	SNP	BP	Effect allele	Other allele	Discovery (N = 1,488)			Combined Sample (N = 4,206)		
					OR	95%CI	*P*	OR	95%CI	*P*
7	rs1800796	22726627	G	C	0.79	[0.62,0.99]	0.0427	0.84	[0.73,0.98]	0.0228
7	rs2069837	22728408	G	T	1.08	[0.83,1.39]	0.5652	1.02	[0.87,1.20]	0.7794
19	rs6508976	41348769	G	T	1.03	[0.84,1.26]	0.7539	0.99	[0.88,1.13]	0.9292
**19**	**rs1800470**	**41353016**	**G**	**A**	**1.37**	**[1.12,1.68]**	**0.0023**	**1.26**	[1.11,1.42]	**0.0004**

The significant SNP is indicated in bold. The *P* value threshold used in the combined sample was 0.05/2 = 0.025.

We performed association analyses for rs1800470 stratified by different DDH types. Our results showed that the significance of rs1800470 was retained only in the severe type, i.e., dislocation of the hips. Conversely, this SNP did not show any significant association with the disease status of DDH for the mild to moderate types (acetabular dysplasia and subluxation of the hips) (Table 3). Furthermore, in case-only analyses, rs1800470 did not show a significant association with the severity of DDH (Supplemental Table S4), and no significant signals were identified in pair-wise gene-by-gene interaction analyses after Bonferroni corrections were applied (Supplemental Table S5).

**Table 3:** Stratification analyses based on different types of DDH for SNPs genotyped in combined samples.

CHR	SNP	BP	A1	Dislocation of the Hips (N = 3,933)		Acetabular Dysplasia (N = 3,202)		Subluxation of the Hips (N = 3,355)	
				OR	*P*	OR	*P*	OR	*P*
7	rs1800796	22726627	G	0.82	0.0138	0.67	0.0963	1.01	0.9234
7	rs2069837	22728408	G	0.99	0.8965	0.81	0.4142	1.22	0.1426
19	rs6508976	41348769	G	0.99	0.8886	1.04	0.8413	1.08	0.5063
**19**	**rs1800470**	**41353016**	**G**	**1.28**	**0.0004**	**1.29**	**0.2030**	**1.24**	**0.0570**

Significant results are highlighted in bold.

### Bioinformatic Analyses

rs1800470 is a common non-synonymous SNP (P [Pro] - L [Leu]) located in exon 7 of *TGFB1*. Population-based data from ExAc^35^ and the 1000 Genomes Project^22^ show that the MAF of the C allele is > 0.4 in both the Chinese population and aggregated populations (similar to our MAF data from the discovery stage). The score of Polyphen2 for this SNP was 0.64, and its biological effect was considered “tolerated”. Functional prediction by SIFT (Sorting Intolerant From Tolerant) classifies this SNP as a “benign” variant. The RegulomeDB score for this SNP is 4, suggesting that this SNP has very limited functional significance (the score system for RegulomeDB ranges from 1 to 6; the higher the score, the lower the functional significance of an SNP).

The sub-network structure of *IL6* and *TGFB1* were explored using STRING, and the interacting genes are shown in Supplemental Figure S2. For *TGFB1*, two other genes from the transforming growth factor gene family, *TGFBR1* and *TGFBR2*, were identified as highly related to our candidate gene. In addition, several genes from the mothers against decapentaplegic homolog (SMAD) gene family, including *SMAD2*, *SMAD3* and *SMAD7*, were also found to interact with *TGFB1*. For *IL-6*, the most related gene identified was the gene encoding its receptor, *IL6R*.

## Discussion

Our study established significant associations between the disease status of DDH and polymorphisms in *TGFB1* (rs1800470) and *IL-6* (rs1800796). Two previous studies attempted to identify relationships between *TGFB1* and *IL-6* with the severe form of DDH^20, 21^. Using data from 28 patients and 20 healthy controls, Kolundžić *et al*. identified association signals of rs1800470 in *TGFB1* and rs1800796 in *IL-6* with severe hip osteoarthritis in adulthood^20^, and using data from 68 cases and 152 controls, Čengić *et al*. reported significant associations between these two SNPs and severe hip osteoarthritis^21^. To some extent, the present study can be considered a replication of these previous analyses; however, our study has several advantages. First, both of these previous studies focused on the severe type of DDH, whereas we recruited DDH patients with different forms of severity. Thus, our study design enabled us to extend our results to a more generalized DDH population instead of only the severe type. In addition, our study design allowed for investigating whether the identified significant SNPs could also serve as an indicator of DDH severity. Our stratification analyses showed that a significant signal of rs1800470 could only be identified in the severe type of DDH (dislocation of the hips), and subsequent analysis focusing on the effects of this SNP and DDH severity showed negative results (rs1800470, OR = 0.99, *P* = 0.9021). This contradiction can be partly explained by differences in sample sizes for the different types of DDH. We recruited a total of 1,064 DDH cases in both stages, and among these subjects, there were 60 cases of acetabular dysplasia, 213 cases of subluxation of hips and 811 cases of dislocation of hip. In stratification analyses, the effect of an SNP might be missed in groups of the less severe types (acetabular dysplasia and subluxation of the hips) due to insufficient statistical power, and the post-hoc statistical power for subgroup of acetabular dysplasia and subluxation of hips were 38% and 55%, respectively. Second, both former studies had very limited sample sizes and suffered from low statistical power, and the ORs of the targeted SNPs were estimated to be very high (6–10). In contrast, our estimation of the OR of rs1800470 was 1.255. This difference can be explained, at least partly, by the different sample sizes used in the two previous studies compared to our study. Given that it is not sufficient to draw conclusions from analyses of only some SNPs^36–40^, our haplotype analyses further corroborated that the SNP rs1800470 was strongly associated with the risk of DDH. Third, both previous studies only considered two SNPs, rs1800470 and rs1800796. Moreover, we selected targeted SNPs using population-based large-scale genotyping data, and the genomic coverage of our study is superior to those of the previous studies.

The significant SNP rs1800470 is a non-synonymous SNP with a high MAF. Although this SNP alters the amino acid sequence of the polypeptide encoded by *TGFB1*, the potential functional consequence remains unclear. One previous study hypothesized that this SNP might affect the peptide export efficiency of TGF-β1^19^. However, this assumption has not been confirmed experimentally. Our bioinformatic analyses based on large-scale databases (SIFT, Polyphen2 and RegulomeDB) indicated that this SNP has very limited functional significance. A possible explanation for the statistical association signal identified in this study for rs1800470 is that this signal might be a surrogate for several rare or low-frequency variants that are enriched around the genomic position of this SNP. Regardless, this hypothesis cannot be directly tested in this study, which was mainly based on selected common SNPs. The other significant SNP rs1800796 which located at the 5’ near gene region also had very limited functional significance according to RegulomeDB (with score of 4). Future studies based on sequencing technology and populations from different ethnic groups with larger sample sizes are needed to validate this result.

Population stratification is a challenge for our study. As a candidate gene-based study, it is unrealistic for us to implement standard procedures, such as genomic control or principle component analysis, requiring genome-wide genotyping data to adjust this potential confounder. Nonetheless, this might be a problem, especially considering that the samples for the discovery and validation stages were recruited from different sites. To adjust for this potential confounder, we restricted our sample recruitment process to ensure that the study subjects had relatively similar genetic backgrounds, even though this might bring a new problem of generalization. In addition, for combined analysis using samples from both the discovery and validation stages, we included a covariate indicating the sample batch to adjust for potential batch effects. Another potential issue is that SNP rs1800470 was not significant in the validation stage (OR = 1.18, *P* = 0.04). We performed a post-hoc power analysis using the sample size (N = 2,781) and the OR information (OR = 1.18) of the validation stage for rs1800470, revealing that the statistical power for detecting this SNP was only approximately 40%. Therefore, insufficient statistical power might have had a strong contribution.

The TGF-β/SMAD signalling pathway plays a very critical role in the expression of genes controlling differentiation in embryonic stem cells^41^. *SMAD3* encodes a transcriptional modulator that binds to the promoter region of many genes that are regulated by TGF-β. As a candidate-gene-based study, we only focused on two genes: *TGFB1* and *IL-6*. However, according to the results of protein-protein interaction analysis, several other genes appear to strongly interact with our candidate genes (Supplemental Figure S2). Among these, *SMAD3*, which encodes a transcriptional modulator that binds to the promoter region of many genes that are regulated by TGF-β, is particularly interesting. The onset and development of DDH might involve additional genes in a systematic manner. In the future, it will be important to include more *TGFB1*-related genes, perhaps an entire pathway, in a genetics study, and the TGF-β/SMAD signalling pathway is a good candidate in this regard.

## Conclusion

In this study, we investigated the potential association of *TGFB1* and *IL-6* with DDH. Our findings support an important role for *TGFB1* and *IL-6* in the onset and development of DDH, though this significant SNP cannot serve as an indicator of DDH severity. Further research is needed to validate the association identified between *IL-6* and DDH.

## Electronic supplementary material

Supplementary Materials
