# Supplementary material for: Genetic association study of common variants in TGFB1 and IL-6 with developmental dysplasia of the hip in Han Chinese population
Source: Sci Rep. 2017 Aug 31;7:10287. doi: 10.1038/s41598-017-11185-1 (PMC5579245; doi:10.1038/s41598-017-11185-1)
Supplement: Supplementary file 1 — Supplementary Materials [file 41598_2017_11185_MOESM1_ESM.pdf]

Title: Genetic association study of common variants in *TGFB1* and *IL-6* with developmental dysplasia of the hip in Han Chinese population

Author names and affiliations: Wenlong Ma [a*], Zhuqing Zha [c*], Ke Chen [a], Honggan Chen [a], Yixin Wu [a], Jianbing Ma [b], Sixiang Zeng [d], Liqiang Zhi [b] and Shuxin Yao [b]

[a] Department of Hip Injury and Disease, Orthopedic Hospital of Henan Province, Qiming South Road No.82, Luoyang, Henan, China;
[b] Department of Joint Surgery, Honghui Hospital, Xi'an Jiaotong University Health Science Center, Xi'an, Shaanxi, China;
[c] The Third Clinical Medical College of Guangzhou University of Traditional Chinese Medicine, Guangzhou, Guangdong, China;
[d] Department of Orthopedics, the First Affiliated Hospital, Xi'an Jiaotong University, Xi'an, Shaanxi, China.

* These authors contributed equally to this work.

Corresponding Author:
Liqiang Zhi, M.D., Department of Joint Surgery, Honghui Hospital, Xi'an Jiaotong University Health Science Center, No.555, Youyi East Road, Xi'an, Shaanxi, China, 710054.
Tel: 86-29-88418011; Fax: 86-29-62818386; E-mail: zhiliqiang2011@126.com

Shuxin Yao, M.D. & Ph.D., Department of Joint Surgery, Honghui Hospital, Xi'an Jiaotong University Health Science Center, No.555, Youyi East Road, Xi'an, Shaanxi, China, 710054.
Tel: 86-29-88418009; Fax: 86-29-62818386; E-mail: popgold@163.com

Supplemental methods

Power Analyses

We conducted comprehensive power analyses for the single marker-based association study using Genetic Power Calculator (http://pngu.mgh.harvard.edu/~purcell/gpc/). For parameter setting, we chose 0.1% as the prevalence of DDH, and we hypothesized that the allele frequency of our risk allele was 0.21, which is the average MAF of the 32 selected markers in the discovery stage. The results of the power analyses showed that for an underlying marker with a relative risk of 1.25, our study design could achieve a statistical power of 0.7996 when the MAFs of the risk marker and tested marker were matched. Thus, our study design was efficient to detect markers with a relative risk greater than or equal to 1.25.

Supplemental Table S1. MAF and Hardy-Weingberg Equilibrium Test results of the 32 selected SNPs.

CHR	SNP	Position	Alleles	Func	Loci	MAF	HWE
7	rs1800796	22726627	C/G	near-gene-5	*IL6*	0.25	0.65
7	rs2069837	22728408	A/G	intron	*IL6*	0.19	0.92
7	rs2066992	22728630	G/T	intron	*IL6*	0.29	1.00
7	rs2069840	22728953	C/G	intron	*IL6*	0.09	1.00
7	rs55998531	22729053	A/G	intron	*IL6*	0.07	0.35
7	rs13306433	22729295	A/G	intron	*IL6*	0.05	0.18
7	rs2069847	22730989	A/G	intron	*IL6*	0.08	1.00
7	rs13306436	22731677	A/G	untranslated-3	*IL6*	0.08	0.56
19	rs1056854	41304090	A/G	coding-synon	*TGFB1*	0.23	0.93
19	rs76567264	41319004	G/T	intron	*TGFB1*	0.08	1.00
19	rs77665081	41320947	A/G	intron	*TGFB1*	0.07	0.62
19	rs147152044	41321198	A/G	intron	*TGFB1*	0.09	1.00
19	rs7257742	41321447	G/T	intron	*TGFB1*	0.36	1.00
19	rs2241719	41323676	A/T	untranslated-3	*TGFB1*	0.28	0.94
19	rs117068651	41326647	A/T	unknown	*TGFB1*	0.07	0.48
19	rs10417924	41327262	C/T	unknown	*TGFB1*	0.15	0.82
19	rs191638973	41327833	C/T	unknown	*TGFB1*	0.06	0.61
19	rs190566789	41332269	C/T	coding-synon	*TGFB1*	0.05	0.32
19	rs8105161	41333726	C/T	intron	*TGFB1*	0.35	0.90
19	rs11466347	41335193	A/G	intron	*TGFB1*	0.27	0.82
19	rs11466345	41337556	A/G	intron	*TGFB1*	0.28	0.83
19	rs6508975	41338654	C/T	intron	*TGFB1*	0.32	0.89
19	rs11466344	41339408	A/G	intron	*TGFB1*	0.06	0.79
19	rs2278422	41339853	C/G	intron	*TGFB1*	0.06	0.43

19	rs10416269	41342943	A/G	intron	*TGFB1*	0.43	0.95
19	rs144114219	41345370	A/G	intron	*TGFB1*	0.07	0.38
19	rs7249191	41345410	C/T	intron	*TGFB1*	0.36	0.70
19	rs116975930	41345578	A/C	intron	*TGFB1*	0.05	0.32
19	rs6508976	41348769	A/G/T	intron	*TGFB1*	0.44	0.86
19	rs1800470	41353016	C/T	missense	*TGFB1*	0.46	0.95
19	rs1800469	41354391	C/T	near-gene-3,near-gene-5	*TGFB1*	0.48	0.95
19	rs4803457	41355454	C/T	intron,near-gene-5	*TGFB1*	0.48	0.86

Supplemental Table S2. Full results of single marker based association analysis using three coding models in discovery stage.

				Additive Model			Dominant Model			Recessive Model		
CHR	SNP	BP	A1	OR	STAT	*P*	OR	STAT	*P*	OR	STAT	*P*
7	rs1800796	22726627	G	0.79	-2.03	0.0427	0.78	-1.70	0.0894	0.59	-1.66	0.0979
7	rs2069837	22728408	G	1.08	0.58	0.5652	1.11	0.67	0.5038	1.00	0.00	0.9996
7	rs2066992	22728630	G	1.07	0.62	0.5334	1.06	0.42	0.6777	1.21	0.70	0.4862
7	rs2069840	22728953	G	0.97	-0.16	0.8741	0.94	-0.31	0.7573	1.45	0.54	0.5894
7	rs55998531	22729053	G	0.78	-1.24	0.2150	0.76	-1.31	0.1892	0.96	-0.04	0.9644
7	rs13306433	22729295	A	0.72	-1.41	0.1592	0.68	-1.53	0.1263	0.97	-0.04	0.9718
7	rs2069847	22730989	A	0.98	-0.08	0.9333	1.02	0.09	0.9290	0.62	-0.65	0.5156
7	rs13306436	22731677	A	1.02	0.12	0.9014	1.05	0.26	0.7959	0.57	-0.58	0.5607
19	rs1056854	41304090	G	0.90	-0.84	0.3993	0.93	-0.53	0.5938	0.72	-1.05	0.2942
19	rs76567264	41319004	T	1.05	0.25	0.7995	1.05	0.23	0.8154	1.15	0.16	0.8690
19	rs77665081	41320947	A	0.90	-0.50	0.6188	0.90	-0.48	0.6292	0.82	-0.22	0.8285
19	rs147152044	41321198	G	0.90	-0.61	0.5390	0.86	-0.79	0.4306	1.61	0.60	0.5466
19	rs7257742	41321447	T	0.91	-0.86	0.3923	0.88	-0.85	0.3959	0.90	-0.48	0.6284
19	rs2241719	41323676	A	0.92	-0.73	0.4680	0.90	-0.71	0.4777	0.90	-0.40	0.6920
19	rs117068651	41326647	T	1.21	0.98	0.3293	1.17	0.74	0.4581	4.16	1.37	0.1709
19	rs10417924	41327262	T	0.98	-0.16	0.8713	1.01	0.03	0.9767	0.78	-0.58	0.5600
19	rs191638973	41327833	T	0.84	-0.83	0.4041	0.84	-0.81	0.4186	0.71	-0.38	0.7041
19	rs190566789	41332269	T	0.86	-0.68	0.4953	0.86	-0.62	0.5348	0.63	-0.47	0.6355

19	rs8105161	41333726	C	0.94	-0.56	0.5745	0.92	-0.55	0.5845	0.93	-0.33	0.7423
19	rs11466347	41335193	T	0.96	-0.35	0.7249	0.93	-0.51	0.6068	1.04	0.13	0.8949
19	rs11466345	41337556	C	0.96	-0.33	0.7447	0.94	-0.45	0.6539	1.02	0.07	0.9439
19	rs6508975	41338654	C	1.03	0.28	0.7782	0.99	-0.07	0.9403	1.19	0.73	0.4641
19	rs11466344	41339408	T	1.13	0.60	0.5492	1.15	0.65	0.5188	0.98	-0.02	0.9868
19	rs2278422	41339853	C	1.05	0.24	0.8123	1.06	0.27	0.7890	0.98	-0.03	0.9769
19	rs10416269	41342943	G	1.04	0.39	0.6937	1.04	0.23	0.8191	1.09	0.43	0.6647
19	rs144114219	41345370	A	0.98	-0.12	0.9006	0.99	-0.03	0.9783	0.69	-0.42	0.6725
19	rs7249191	41345410	C	1.00	0.04	0.9667	0.98	-0.15	0.8785	1.07	0.31	0.7599
19	rs116975930	41345578	A	0.97	-0.13	0.8974	0.94	-0.24	0.8072	1.80	0.51	0.6115
19	rs6508976	41348769	G	1.03	0.31	0.7539	1.01	0.08	0.9340	1.09	0.45	0.6498
19	rs1800470	41353016	G	1.37	3.05	0.0023	1.46	2.28	0.0224	1.58	2.69	0.0071
19	rs1800469	41354391	A	0.97	-0.34	0.7337	1.00	0.03	0.9784	0.90	-0.60	0.5517
19	rs4803457	41355454	T	0.95	-0.53	0.5932	0.92	-0.49	0.6264	0.94	-0.38	0.7024

Supplemental Table S3. Results of the haplotype analyses.

LOCUS	HAPLOTYPE	F_A	F_U	CHISQ	DF	*P*	SNPS
H1	OMNIBUS	-	-	1.463	2	0.4812	rs7257742|rs2241719
H1	TA	0.268	0.284	0.696	1	0.4042	
H1	TT	0.088	0.077	1.028	1	0.3107	
H1	GT	0.644	0.640	0.043	1	0.8348	
H2	OMNIBUS	-	-	1.767	2	0.4133	rs11466347|rs11466345
H2	TC	0.263	0.265	0.008	1	0.9286	
H2	CC	0.029	0.020	1.766	1	0.1839	
H2	CT	0.708	0.715	0.120	1	0.7286	
H3	OMNIBUS	-	-	118.700	2	**1.65×10^{-26}**	**rs6508976|rs1800470**
H3	GG	0.443	0.437	0.090	1	0.7643	
H3	TG	0.081	0.008	115.200	1	7.12×10^{-27}	
H3	TA	0.476	0.555	14.060	1	0.0002	
H4	OMNIBUS	-	-	1.611	2	0.4468	rs1800469|rs4803457
H4	AT	0.469	0.472	0.022	1	0.8832	
H4	GT	0.015	0.009	1.611	1	0.2044	
H4	GC	0.516	0.519	0.014	1	0.9074	

Significant results were highlighted in bold.

Supplemental Table S4. Results of the association analyses between genotyped markers in combined samples and the severity of DDH.

CHR	SNP	BP	Effect Allele	Other Allele	OR	95%CI	STAT	*P*
7	rs1800796	22726627	G	C	0.85	[0.68，1.07]	-1.36	0.1755
7	rs2069837	22728408	G	T	0.88	[0.69，1.13]	-1.00	0.3175
19	rs6508976	41348769	G	T	0.90	[0.74，1.09]	-1.06	0.2881
19	rs1800470	41353016	G	**A**	0.99	[0.81，1.20]	-0.12	0.9021

Supplemental Table S5. Summarized results of gene by gene interaction analyses.

SNP1	CHR1	GENE1	SNP2	CHR2	GENE2	inter.OR	inter.Z	inter.P
rs13306436	7	*IL6*	rs11466345	19	*TGFB1*	2.16	2.46	0.0137
rs13306433	7	*IL6*	rs11466347	19	*TGFB1*	2.47	2.22	0.0265
rs13306433	7	*IL6*	rs11466345	19	*TGFB1*	2.41	2.17	0.0302
rs13306436	7	*IL6*	rs11466347	19	*TGFB1*	1.89	2.07	0.0386
rs13306436	7	*IL6*	rs6508975	19	*TGFB1*	1.86	2.04	0.0413
rs2066992	7	*IL6*	rs116975930	19	*TGFB1*	0.42	-1.98	0.0472
rs2069847	7	*IL6*	rs2241719	19	*TGFB1*	0.55	-1.92	0.0543
rs13306436	7	*IL6*	rs6508976	19	*TGFB1*	1.60	1.74	0.0810
rs2069847	7	*IL6*	rs11466347	19	*TGFB1*	1.66	1.66	0.0973
rs2069847	7	*IL6*	rs6508975	19	*TGFB1*	1.58	1.64	0.1011
rs2069840	7	*IL6*	rs10417924	19	*TGFB1*	0.58	-1.55	0.1211
rs13306436	7	*IL6*	rs1800470	19	*TGFB1*	1.52	1.54	0.1244
rs13306436	7	*IL6*	rs117068651	19	*TGFB1*	2.10	1.53	0.1258
rs2069847	7	*IL6*	rs116975930	19	*TGFB1*	0.32	-1.51	0.1299
rs13306433	7	*IL6*	rs6508975	19	*TGFB1*	1.78	1.51	0.1301
rs13306436	7	*IL6*	rs144114219	19	*TGFB1*	0.34	-1.51	0.1308
rs2069840	7	*IL6*	rs1056854	19	*TGFB1*	1.53	1.51	0.1314
rs55998531	7	*IL6*	rs11466344	19	*TGFB1*	2.30	1.49	0.1372
rs2069847	7	*IL6*	rs8105161	19	*TGFB1*	1.54	1.48	0.1393
rs13306433	7	*IL6*	rs8105161	19	*TGFB1*	1.68	1.48	0.1401
rs13306433	7	*IL6*	rs2278422	19	*TGFB1*	0.34	-1.46	0.1445
rs13306436	7	*IL6*	rs8105161	19	*TGFB1*	1.52	1.46	0.1449
rs1800796	7	*IL6*	rs76567264	19	*TGFB1*	1.60	1.45	0.1481
rs2069847	7	*IL6*	rs11466344	19	*TGFB1*	2.07	1.43	0.1521
rs2069847	7	*IL6*	rs11466345	19	*TGFB1*	1.52	1.40	0.1625
rs13306436	7	*IL6*	rs11466344	19	*TGFB1*	2.11	1.37	0.1707
rs2069847	7	*IL6*	rs10417924	19	*TGFB1*	1.53	1.35	0.1761
rs2069837	7	*IL6*	rs116975930	19	*TGFB1*	0.54	-1.35	0.1782
rs13306436	7	*IL6*	rs1800469	19	*TGFB1*	1.40	1.34	0.1806
rs2069840	7	*IL6*	rs144114219	19	*TGFB1*	1.86	1.33	0.1844
rs2069840	7	*IL6*	rs6508976	19	*TGFB1*	1.38	1.32	0.1884
rs55998531	7	*IL6*	rs147152044	19	*TGFB1*	0.47	-1.31	0.1892
rs55998531	7	*IL6*	rs2241719	19	*TGFB1*	0.65	-1.31	0.1893
rs55998531	7	*IL6*	rs144114219	19	*TGFB1*	2.07	1.31	0.1900
rs55998531	7	*IL6*	rs10417924	19	*TGFB1*	1.59	1.30	0.1942
rs1800796	7	*IL6*	rs116975930	19	*TGFB1*	0.58	-1.29	0.1956
rs2069837	7	*IL6*	rs7257742	19	*TGFB1*	1.28	1.27	0.2026
rs2069840	7	*IL6*	rs1800470	19	*TGFB1*	1.38	1.27	0.2048
rs13306436	7	*IL6*	rs116975930	19	*TGFB1*	0.38	-1.24	0.2136
rs55998531	7	*IL6*	rs6508975	19	*TGFB1*	1.43	1.22	0.2222

rs55998531	7	*IL6*	rs11466345	19	*TGFB1*	1.44	1.22	0.2233
rs13306436	7	*IL6*	rs4803457	19	*TGFB1*	1.36	1.21	0.2266
rs55998531	7	*IL6*	rs7249191	19	*TGFB1*	1.44	1.21	0.2269
rs55998531	7	*IL6*	rs116975930	19	*TGFB1*	0.34	-1.21	0.2272
rs13306433	7	*IL6*	rs116975930	19	*TGFB1*	0.24	-1.20	0.2309
rs2066992	7	*IL6*	rs11466347	19	*TGFB1*	1.24	1.18	0.2375
rs2069847	7	*IL6*	rs191638973	19	*TGFB1*	0.53	-1.16	0.2453
rs1800796	7	*IL6*	rs117068651	19	*TGFB1*	1.47	1.15	0.2494
rs55998531	7	*IL6*	rs11466347	19	*TGFB1*	1.40	1.15	0.2515
rs1800796	7	*IL6*	rs11466344	19	*TGFB1*	1.46	1.12	0.2606
rs55998531	7	*IL6*	rs4803457	19	*TGFB1*	1.37	1.12	0.2617
rs13306436	7	*IL6*	rs77665081	19	*TGFB1*	1.63	1.11	0.2666
rs13306436	7	*IL6*	rs10417924	19	*TGFB1*	0.69	-1.09	0.2754
rs1800796	7	*IL6*	rs190566789	19	*TGFB1*	1.48	1.00	0.3161
rs2069837	7	*IL6*	rs4803457	19	*TGFB1*	0.83	-1.00	0.3180
rs2069837	7	*IL6*	rs11466344	19	*TGFB1*	1.40	0.98	0.3296
rs55998531	7	*IL6*	rs1800469	19	*TGFB1*	1.32	0.97	0.3299
rs2069847	7	*IL6*	rs1800469	19	*TGFB1*	0.79	-0.96	0.3389
rs2066992	7	*IL6*	rs191638973	19	*TGFB1*	1.38	0.94	0.3472
rs1800796	7	*IL6*	rs1800470	19	*TGFB1*	0.85	-0.94	0.3476
rs1800796	7	*IL6*	rs77665081	19	*TGFB1*	1.39	0.92	0.3572
rs2069840	7	*IL6*	rs116975930	19	*TGFB1*	0.57	-0.91	0.3630
rs2069837	7	*IL6*	rs191638973	19	*TGFB1*	1.43	0.89	0.3752
rs2069837	7	*IL6*	rs2241719	19	*TGFB1*	1.20	0.88	0.3791
rs13306436	7	*IL6*	rs2278422	19	*TGFB1*	0.63	-0.86	0.3895
rs2069840	7	*IL6*	rs7249191	19	*TGFB1*	1.24	0.86	0.3918
rs2069837	7	*IL6*	rs8105161	19	*TGFB1*	0.85	-0.85	0.3945
rs2069847	7	*IL6*	rs7257742	19	*TGFB1*	0.80	-0.83	0.4077
rs2069837	7	*IL6*	rs11466345	19	*TGFB1*	0.85	-0.83	0.4079
rs2069837	7	*IL6*	rs1800470	19	*TGFB1*	0.85	-0.83	0.4080
rs2069847	7	*IL6*	rs147152044	19	*TGFB1*	0.67	-0.83	0.4084
rs1800796	7	*IL6*	rs2278422	19	*TGFB1*	0.77	-0.82	0.4118
rs2069840	7	*IL6*	rs7257742	19	*TGFB1*	1.21	0.80	0.4212
rs2069847	7	*IL6*	rs76567264	19	*TGFB1*	0.71	-0.80	0.4226
rs2069847	7	*IL6*	rs117068651	19	*TGFB1*	1.43	0.79	0.4267
rs55998531	7	*IL6*	rs117068651	19	*TGFB1*	0.66	-0.78	0.4327
rs1800796	7	*IL6*	rs1056854	19	*TGFB1*	1.16	0.78	0.4383
rs2069847	7	*IL6*	rs4803457	19	*TGFB1*	0.83	-0.77	0.4438
rs2069847	7	*IL6*	rs77665081	19	*TGFB1*	0.67	-0.76	0.4451
rs55998531	7	*IL6*	rs77665081	19	*TGFB1*	0.61	-0.76	0.4501
rs13306433	7	*IL6*	rs10416269	19	*TGFB1*	0.79	-0.73	0.4675
rs2066992	7	*IL6*	rs2241719	19	*TGFB1*	1.14	0.72	0.4705
rs2069837	7	*IL6*	rs1800469	19	*TGFB1*	0.88	-0.72	0.4718
rs2069847	7	*IL6*	rs10416269	19	*TGFB1*	1.19	0.67	0.5026

rs1800796	7	*IL6*	rs7249191	19	*TGFB1*	0.89	-0.67	0.5029
rs1800796	7	*IL6*	rs191638973	19	*TGFB1*	1.27	0.66	0.5118
rs13306433	7	*IL6*	rs144114219	19	*TGFB1*	0.67	-0.65	0.5130
rs2066992	7	*IL6*	rs6508976	19	*TGFB1*	1.12	0.65	0.5160
rs2069837	7	*IL6*	rs117068651	19	*TGFB1*	1.26	0.64	0.5253
rs13306436	7	*IL6*	rs2241719	19	*TGFB1*	0.83	-0.61	0.5420
rs2066992	7	*IL6*	rs11466345	19	*TGFB1*	1.12	0.61	0.5432
rs2069837	7	*IL6*	rs6508975	19	*TGFB1*	0.89	-0.61	0.5447
rs13306436	7	*IL6*	rs147152044	19	*TGFB1*	1.30	0.60	0.5492
rs1800796	7	*IL6*	rs6508975	19	*TGFB1*	1.11	0.59	0.5584
rs13306433	7	*IL6*	rs2241719	19	*TGFB1*	0.80	-0.58	0.5588
rs2069847	7	*IL6*	rs7249191	19	*TGFB1*	1.16	0.58	0.5594
rs13306433	7	*IL6*	rs1800470	19	*TGFB1*	0.82	-0.56	0.5724
rs2069837	7	*IL6*	rs77665081	19	*TGFB1*	0.79	-0.56	0.5751
rs2069840	7	*IL6*	rs11466347	19	*TGFB1*	0.85	-0.56	0.5760
rs2069840	7	*IL6*	rs191638973	19	*TGFB1*	0.75	-0.55	0.5812
rs55998531	7	*IL6*	rs76567264	19	*TGFB1*	0.73	-0.55	0.5824
rs13306433	7	*IL6*	rs4803457	19	*TGFB1*	0.85	-0.54	0.5893
rs2066992	7	*IL6*	rs8105161	19	*TGFB1*	0.91	-0.54	0.5898
rs55998531	7	*IL6*	rs6508976	19	*TGFB1*	1.18	0.53	0.5959
rs1800796	7	*IL6*	rs7257742	19	*TGFB1*	1.10	0.51	0.6071
rs13306433	7	*IL6*	rs76567264	19	*TGFB1*	1.44	0.51	0.6076
rs1800796	7	*IL6*	rs11466347	19	*TGFB1*	1.10	0.50	0.6185
rs2069837	7	*IL6*	rs1056854	19	*TGFB1*	0.89	-0.49	0.6225
rs13306433	7	*IL6*	rs190566789	19	*TGFB1*	0.71	-0.49	0.6242
rs2069837	7	*IL6*	rs2278422	19	*TGFB1*	0.83	-0.49	0.6252
rs13306433	7	*IL6*	rs147152044	19	*TGFB1*	1.35	0.47	0.6356
rs13306433	7	*IL6*	rs117068651	19	*TGFB1*	0.74	-0.47	0.6382
rs2066992	7	*IL6*	rs117068651	19	*TGFB1*	0.86	-0.46	0.6426
rs55998531	7	*IL6*	rs1056854	19	*TGFB1*	1.17	0.46	0.6460
rs55998531	7	*IL6*	rs2278422	19	*TGFB1*	1.33	0.46	0.6485
rs13306433	7	*IL6*	rs77665081	19	*TGFB1*	0.66	-0.45	0.6500
rs1800796	7	*IL6*	rs144114219	19	*TGFB1*	0.86	-0.45	0.6524
rs13306433	7	*IL6*	rs1800469	19	*TGFB1*	0.88	-0.44	0.6567
rs2069847	7	*IL6*	rs2278422	19	*TGFB1*	0.77	-0.44	0.6569
rs2069840	7	*IL6*	rs147152044	19	*TGFB1*	1.19	0.44	0.6606
rs13306433	7	*IL6*	rs1056854	19	*TGFB1*	1.20	0.44	0.6618
rs2069840	7	*IL6*	rs190566789	19	*TGFB1*	1.27	0.43	0.6636
rs1800796	7	*IL6*	rs10416269	19	*TGFB1*	0.93	-0.43	0.6639
rs2066992	7	*IL6*	rs7257742	19	*TGFB1*	1.07	0.43	0.6643
rs2066992	7	*IL6*	rs77665081	19	*TGFB1*	0.88	-0.42	0.6757
rs2069840	7	*IL6*	rs10416269	19	*TGFB1*	1.11	0.42	0.6771
rs13306436	7	*IL6*	rs76567264	19	*TGFB1*	1.23	0.40	0.6917
rs2066992	7	*IL6*	rs11466344	19	*TGFB1*	1.13	0.40	0.6921

rs2069840	7	*IL6*	rs8105161	19	*TGFB1*	1.10	0.38	0.7028
rs2069837	7	*IL6*	rs144114219	19	*TGFB1*	1.14	0.38	0.7043
rs13306433	7	*IL6*	rs7249191	19	*TGFB1*	0.87	-0.37	0.7129
rs2069847	7	*IL6*	rs144114219	19	*TGFB1*	0.85	-0.36	0.7214
rs1800796	7	*IL6*	rs1800469	19	*TGFB1*	1.06	0.33	0.7388
rs2066992	7	*IL6*	rs10416269	19	*TGFB1*	1.05	0.32	0.7487
rs2069837	7	*IL6*	rs10417924	19	*TGFB1*	1.09	0.32	0.7522
rs13306433	7	*IL6*	rs7257742	19	*TGFB1*	1.10	0.30	0.7646
rs2066992	7	*IL6*	rs7249191	19	*TGFB1*	1.05	0.30	0.7650
rs2069847	7	*IL6*	rs1056854	19	*TGFB1*	1.09	0.29	0.7702
rs13306436	7	*IL6*	rs7257742	19	*TGFB1*	1.09	0.29	0.7706
rs1800796	7	*IL6*	rs147152044	19	*TGFB1*	1.10	0.29	0.7748
rs2069837	7	*IL6*	rs76567264	19	*TGFB1*	0.90	-0.28	0.7760
rs55998531	7	*IL6*	rs10416269	19	*TGFB1*	1.08	0.26	0.7943
rs2066992	7	*IL6*	rs6508975	19	*TGFB1*	0.96	-0.26	0.7963
rs2069840	7	*IL6*	rs2278422	19	*TGFB1*	0.88	-0.25	0.8037
rs1800796	7	*IL6*	rs8105161	19	*TGFB1*	0.96	-0.23	0.8146
rs13306436	7	*IL6*	rs190566789	19	*TGFB1*	0.86	-0.23	0.8210
rs1800796	7	*IL6*	rs10417924	19	*TGFB1*	0.95	-0.23	0.8210
rs1800796	7	*IL6*	rs4803457	19	*TGFB1*	1.04	0.22	0.8230
rs55998531	7	*IL6*	rs1800470	19	*TGFB1*	1.07	0.22	0.8248
rs55998531	7	*IL6*	rs8105161	19	*TGFB1*	1.07	0.22	0.8257
rs13306436	7	*IL6*	rs191638973	19	*TGFB1*	0.91	-0.22	0.8262
rs2066992	7	*IL6*	rs2278422	19	*TGFB1*	0.93	-0.22	0.8265
rs2069847	7	*IL6*	rs1800470	19	*TGFB1*	0.94	-0.22	0.8270
rs2066992	7	*IL6*	rs76567264	19	*TGFB1*	1.07	0.20	0.8381
rs2066992	7	*IL6*	rs190566789	19	*TGFB1*	1.08	0.19	0.8458
rs55998531	7	*IL6*	rs190566789	19	*TGFB1*	1.12	0.19	0.8514
rs2069837	7	*IL6*	rs190566789	19	*TGFB1*	1.08	0.18	0.8537
rs13306436	7	*IL6*	rs10416269	19	*TGFB1*	1.05	0.18	0.8541
rs2066992	7	*IL6*	rs4803457	19	*TGFB1*	0.97	-0.18	0.8600
rs2069840	7	*IL6*	rs1800469	19	*TGFB1*	0.96	-0.18	0.8606
rs13306433	7	*IL6*	rs11466344	19	*TGFB1*	1.12	0.17	0.8689
rs55998531	7	*IL6*	rs7257742	19	*TGFB1*	0.96	-0.15	0.8795
rs1800796	7	*IL6*	rs2241719	19	*TGFB1*	1.03	0.13	0.8936
rs13306436	7	*IL6*	rs7249191	19	*TGFB1*	1.04	0.13	0.8947
rs13306436	7	*IL6*	rs1056854	19	*TGFB1*	1.04	0.13	0.8970
rs2069837	7	*IL6*	rs7249191	19	*TGFB1*	1.02	0.12	0.9040
rs2069837	7	*IL6*	rs10416269	19	*TGFB1*	0.98	-0.12	0.9060
rs2066992	7	*IL6*	rs144114219	19	*TGFB1*	0.97	-0.12	0.9082
rs2069840	7	*IL6*	rs11466345	19	*TGFB1*	0.97	-0.12	0.9082
rs2069837	7	*IL6*	rs11466347	19	*TGFB1*	0.98	-0.11	0.9087
rs2069840	7	*IL6*	rs76567264	19	*TGFB1*	1.06	0.11	0.9088
rs2066992	7	*IL6*	rs147152044	19	*TGFB1*	1.03	0.11	0.9093

rs2066992	7	*IL6*	rs1800469	19	*TGFB1*	0.98	-0.10	0.9166
rs13306433	7	*IL6*	rs191638973	19	*TGFB1*	1.06	0.10	0.9166
rs2069837	7	*IL6*	rs6508976	19	*TGFB1*	1.02	0.10	0.9176
rs2069840	7	*IL6*	rs117068651	19	*TGFB1*	1.04	0.10	0.9221
rs1800796	7	*IL6*	rs6508976	19	*TGFB1*	0.98	-0.10	0.9227
rs2069840	7	*IL6*	rs4803457	19	*TGFB1*	0.98	-0.09	0.9250
rs1800796	7	*IL6*	rs11466345	19	*TGFB1*	1.02	0.09	0.9286
rs13306433	7	*IL6*	rs10417924	19	*TGFB1*	0.97	-0.08	0.9324
rs2069840	7	*IL6*	rs77665081	19	*TGFB1*	0.96	-0.08	0.9334
rs2069837	7	*IL6*	rs147152044	19	*TGFB1*	1.03	0.08	0.9400
rs2069840	7	*IL6*	rs6508975	19	*TGFB1*	1.02	0.06	0.9490
rs2066992	7	*IL6*	rs1056854	19	*TGFB1*	1.01	0.06	0.9508
rs2066992	7	*IL6*	rs10417924	19	*TGFB1*	1.01	0.06	0.9530
rs2069847	7	*IL6*	rs190566789	19	*TGFB1*	0.97	-0.05	0.9626
rs13306433	7	*IL6*	rs6508976	19	*TGFB1*	0.99	-0.04	0.9683
rs2069847	7	*IL6*	rs6508976	19	*TGFB1*	0.99	-0.03	0.9759
rs2069840	7	*IL6*	rs2241719	19	*TGFB1*	1.00	-0.02	0.9852
rs2069840	7	*IL6*	rs11466344	19	*TGFB1*	0.99	-0.02	0.9876
rs2066992	7	*IL6*	rs1800470	19	*TGFB1*	1.00	-0.02	0.9877
rs55998531	7	*IL6*	rs191638973	19	*TGFB1*	1.01	0.01	0.9921

P value threshold used for this analysis was 0.05/192≈0.00026.

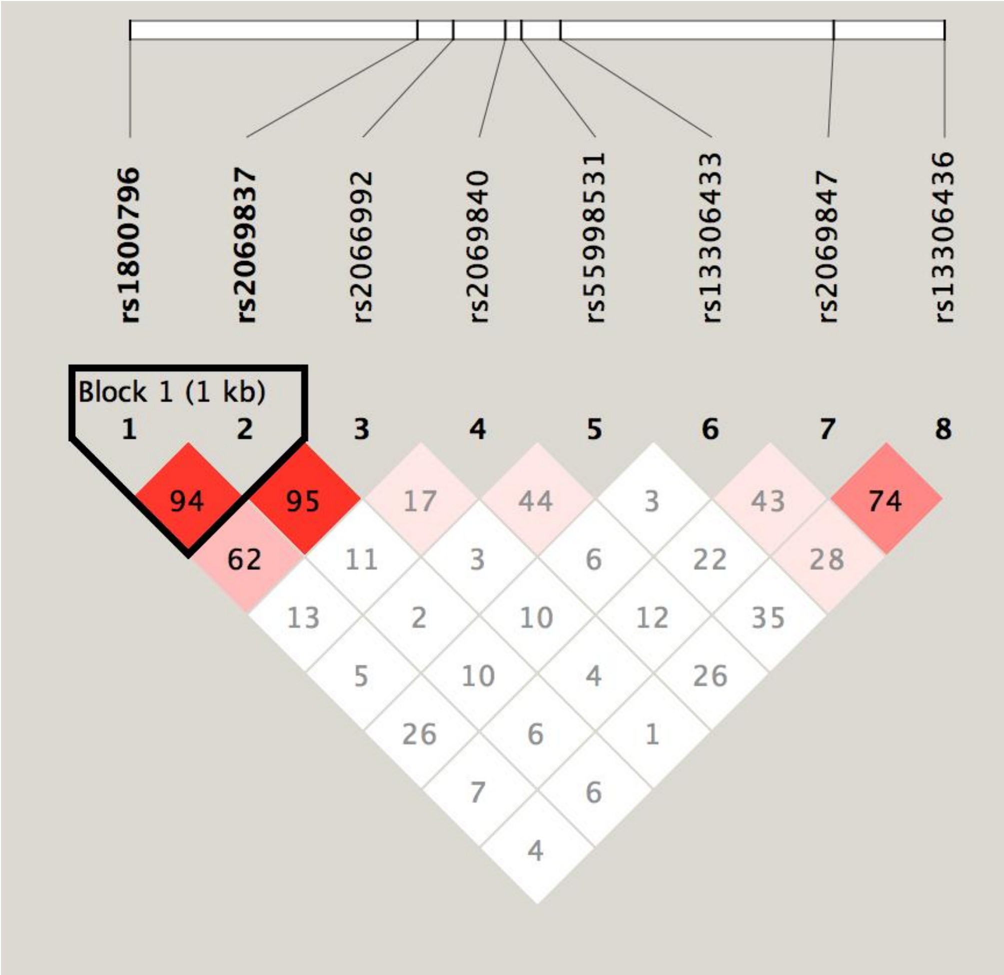

Supplemental Figure S1. LD structure of gene *IL-6* using the genotype data of discovery stage.

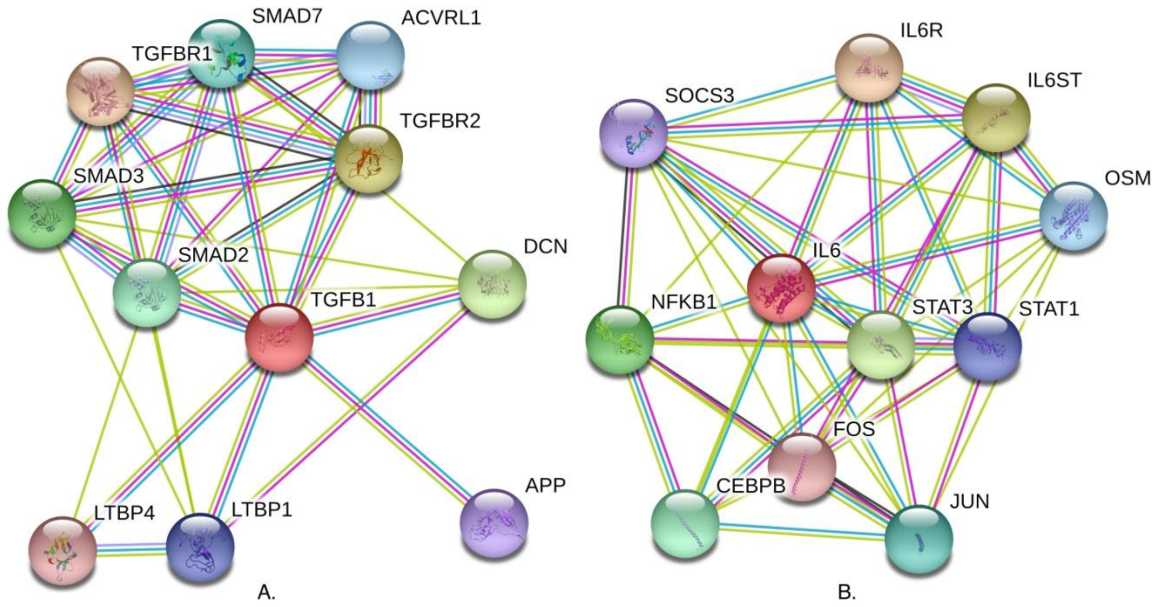

Supplemental Figure S2. Sub-network structure of gene *TGFB1*(A) and *IL-6*(B).
